# Value of Provoked or Spontaneous Flank Pain in Men with Febrile Urinary Tract Infections

**DOI:** 10.3390/antibiotics3020155

**Published:** 2014-04-14

**Authors:** Franck Bruyère, Joseph-Alain Ruimy, Louis Bernard, Raphael Elfassi, Olivier Boyer, Fabrice Amann, Paul Meria

**Affiliations:** 1 Urology Department, University Hospital of Tours, Loire Valley 37044, France; 2 University Francois Rabelais de Tours, PRES Centre Val de Loire Université, Tours 37000, France; E-Mail: Louis.bernard@chu-tours.fr; 3 General Practitioner, 15 rue Jean Baptiste Berlier, Paris 75013, France; E-Mails: joseph.ruimy@spherescare.fr (J.-A.R.); raphael.elfassi@gmail.com (R.E.); olboy92@gmail.com (O.B.); dr.fabrice.amann@ump.fr (F.A.); 4 Infectious Disease Department, University Hospital of Tours, Loire Valley 37044, France; 5 Urology Department, Saint Louis Hospital, Paris 75010, France; E-Mail: paul.meria@sls-aphp.fr

**Keywords:** prostatitis, clinical, general practitioner, network, ambulatory

## Abstract

Background: Our objective was to identify the clinical, laboratory and radiological characteristics of febrile urinary tract infections (UTI) in men and to focus on the value of flank pain in these men managed in an ambulatory care system. Methods: A network was designed to manage men with febrile UTI without hospitalization according to an algorithm designed with different specialists. The patients’ characteristics were prospectively recorded and each patient was followed up until completely cured. We artificially divided patients into two groups. Group 1: men without flank pain diagnosed as prostatitis and a second group (Group 2) of men with flank pain or provoked flank pain more likely to have a pyelonephritis. Groups were compared to find arguments to differentiate prostatitis to pyelonephritis. Results: 350 men were included in the study, half of these men reported urinary symptoms (dysuria, urgency and burning urination). The negative predictive values of the nitrite and leukocytes test were poor alone or in combination. The renal ultrasound was never informative. None of the patients failed to respond to the treatment. No difference was found between groups. Conclusions: Laboratory test results and radiological features had a poor predictive value. Men with suspected pyelonephritis did not evolve differently from those with suspected prostatitis. Monitoring and treatment of men with febrile UTI does not seem to depend on the existence of a pyelonephritis suspected after the presence of a lumbar pain. Ambulatory management of febrile UTI is feasible and safe, requiring an efficient network for patient’s surveillance.

## 1. Introduction

Acute genitourinary tract infections such as acute prostatitis (AP) represent a significant problem in urological practice. Unfortunately, very few data have been published regarding this subject. In the U.S., AP is the most common urological diagnosis in men under 50 years of age, representing 8% of consultations [1]. Antibiotic treatment of AP is recommended, but abscess formation has been described, which may have devastating consequences. It is now acknowledged that the vast majority of febrile urinary infections in men are to be considered as acute prostatitis; pyelonephritis remains exceptional in men. Flank pain, spontaneous or provoked, is the clinical sign that orients the diagnosis toward a pyelonephritis. Unfortunately, no pathognomonic clinical, biological or radiological signs prove the diagnosis. We analyzed 251 cases of febrile urinary infections in men treated in a medical network; all of the data were prospectively recorded by a data manager and were analyzed retrospectively.

## 2. Methods

A network was designed to include general practitioners (GP), private biological departments and private radiological departments to manage febrile urinary infections without hospitalization. Each patient was managed “at home” by scheduling clinical examinations, laboratory tests and imaging using the network. All of the GPs involved in the group were educated on how to use an algorithm initially built with specialists and were asked to enter information into a database. Treatments were defined as successful if symptoms disappeared in less than 72 h and if no recurrence was observed within one month. Herein, we present the entire clinical, laboratory, radiological and bacteriological data for men with acute febrile urinary infection treated between May 2005 and December 2012 as part of the SphereS. We analyzed 350 men who presented with febrile urinary infection. The data were collected prospectively in a customized database and were retrospectively analyzed. We included all men who were recruited by their GP because of suspicion of a febrile urinary infection (fever and urinary symptoms); however, men with suspected urethritis (urethral flow and pain without fever) were excluded from the study. Once enrolled in the study, none of the men were excluded from the final analysis. We artificially divided patients into two groups. Group 1: men without flank pain diagnosed as prostatitis and a second group (Group 2) of men with flank pain or provoked flank pain more likely to have a pyelonephritis. Groups were compared to find arguments to differentiate prostatitis to pyelonephritis.

### Statistical Analysis

Continuous variables were reported as the mean value plus or minus the standard deviation. The sensitivity, specificity and positive and negative predictive values were calculated for several variables. We used chi-square analysis and alternatively, when indicated, Fisher’s exact test and Wilcoxon for non-parametric comparison of means. The significance of the difference between groups was estimated by way of a two-tailed Student’s *t*-test.

## 3. Results and Discussion

Three hundred and fifty men were enrolled in the study. The patient characteristics are summarized in Table 1.

**Table 1:** Patient characteristics of 350 men with febrile urinary infection. Group 1: no flank pain; group 2: provoked or spontaneous flank pain.

characteristics	Total	Group 1 (*n* = 308)	Group 2 (*n* = 42)	*p* value
Mean age ± SD years	60.2 ± 16.2	61.0 ± 15.8	54.8 ± 18.2	0.04
History of UTI (%)	82 (23.4%)	79 (25.6%)	3 (7.1%)	0.02
Diabetes (%)	22 (6.3%)	18 (5.8%)	4 (9.5%)	ns
Known prostate enlargement (%)	37 (10.5%)	36 (11.6%)	1 (2.3%)	ns
Flank pain	18 (5.1%)	0 (%)	18 (42.8%)	<0.0001
Provoked flank pain	39 (11.1%)	0 (0%)	39 (92.8%)	
Macroscopic hematuria	71 (20.3%)	62 (20.1%)	6 (21.4%)	ns
Flu-like symptoms	29 (8.3%)	24 (7.8%)	5 (11.9%)	ns
Urinary bother	178 (50.8%)	159 (51.6%)	19 (45.3%)	ns
painful DRE	15 (4.3%)	14 (4.5%)	1 (2.3%)	ns

Abbreviations: SD, standard deviation; UTI, urinary tract infection; DRE, digital rectal examination; ns, non significant.

All men were referred to their GP because of suspicion of febrile acute urinary tract infection. All men were referred because of fever higher than 38 °C, and half of these men reported urinary symptoms (dysuria, urgency and burning urination).

A total of 289 (82.6%) patients had neutrophil counts exceeding 8000/mm^3^ (Table 2).

No difference was found between groups in terms of urine dipstick results. Among patients with urine dipsticks showing negative for leukocytes and nitrite, only 5.5% had a negative urine culture, 82.8% tested positive for *E. coli*, and 5.7% tested positive for *Proteus mirabilis*. The sensitivity of the nitrite test was 67%, and the specificity was 57%; the sensitivity of the leukocyte test was 94.3%, and the specificity was 5.8%. The addition of the of the nitrite test results marginally improved the sensibility of the leukocyte test; however, the specificity remained low. The positive predictive value of a positive nitrite test and of the combination of a positive nitrite and a positive leukocyte test were respectively 90.1% and 86.1%. The negative predictive values of the nitrite and leukocytes test were poor alone or in combination.

The urine dipstick was positive for leukocytes and nitrite in 97.9% of patients and was negative for both in 2.1% of the cases of positive urine culture (Table 3).

**Table 2:** Laboratory test results for men with acute urinary infection.

Laboratory test	Total	Group 1 (*n* = 308)	Group 2 (*n* = 42)	*p* value
Leukocytes (10^3^/mm^3^)	14.4 ± 4.9	14.7 ± 5.0	14.2 ± 4.3	ns
Neutrophils (10^3^/mm^3^)	12.1 ± 4.6	12.2 ± 4.6	11.6 ± 4.1	ns
Creatinine (µmol/L)	102 ± 24	102 ± 25	97 ± 20	ns
Creatinine clearance (mL/min)	85 ± 25	84 ± 24	95 ± 32	ns
C reactive protein (mg/L)	100 ± 83	99 ± 83	104 ± 83	ns
Urinary leukocytes (mean ± SD)/mm^3^	525 ± 820	551 ± 860	337 ± 379	0.01
Urinary red blood cells	143 ± 312	142 ± 302	152 ± 385	ns
Positive urinary bacterial culture	298 (85.1%)	268 (87.0%)	30 (71.4%)	ns
**Bacteriological findings**				0.04
*Escherichia coli*	247 (82.8%)	230 (85.8%)	17 (56.6%)		
*Proteus*	17 (5.7%)	17 (6.3%)	0 (0.0%)		
*Klebsiella*	8 (2.6%)	6 (2.2%)	2 (6.6%)		
*Citrobacter*	9 (3.0%)	8 (3.0%)	1 (3.3%)		
*Enterococcus*	5 (1.6%)	4 (1.5%)	1 (3.3%)		
*Enterobacter*	4 (1.3%)	4 (1.5%)	0 (0.0%)		
*Morganella*	4 (1.3%)	3 (1.1%)	1 (3.3%)		
*Staphylococcus* sp*.*	3 (1.0%)	3 (1.1%)	0 (0.0%)		
*Haemophilus*	1 (0.3%)	0 (0.0%)	1 (3.3%)		

Abbreviations: ns, non significant.

**Table 3:** Results of the urine dipstick test.

Diagnostic test, result (no. of patients with results)	Culture result, no. (%)	
	positive (*n* = 298)	negative (*n* = 52)
Nitrite		
positive (223)	201 (67.4)	22 (42.3)
negative (127)	97 (32.5)	30 (57.6)
Leukocytes		
positive (330)	281 (94.3)	49 (94.2)
negative (20)	17 (5.7)	3 (5.8)
Nitrite, positive		
Leukocytes positive (339)	292 (97.9)	47 (90.7)
leukocytes negative (11)	6 (2.1)	5 (9.6)
Nitrite, negative		
Leukocytes positive (296)	247 (82.9)	49 (94.2)
Leukocytes negative (54)	51 (17.1)	3 (5.8)

Twenty four patients (6.8%) had a postvoiding volume greater than 80 mL. In the remaining cases, the renal ultrasound was not informative (Table 4).

Two cases of renal dilation were found; however, only one patient exhibited pain in his flank. Stones were more often found in patients with spontaneous or provoked flank pain (*p* = 0.011).

**Table 4:** Ultrasound findings.

	Total (*n* = 350)	Group 1 (*n* = 308)	Group 2 (*n* = 42)	*p* Value
Prostate volume mL (mean ± SD)	43 ± 23	43 ± 21	38 ± 34	ns
Post voiding urinary volume	46 ± 112	47 ± 116	30 ± 54	ns
**Other comments:**				
Prostatic calcifications	17 (50.1%)	16 (55%)	1 (25%)	ns
Epididymitis	2 (5.9%)	1 (3%)	1 (25%)	ns
Prostate nodule	1 (2.9%)	1 (3%)	0	ns
Bladder tumor	2 (5.8%)	2 (7%)	0	ns
Prostatitis	12 (35.1%)	10 (31%)	2 (50%)	ns
Comments on kidney (*n* = 204)				
Angioma	1 (0.4%)	1 (0.5%)	0 (0%)	ns
Stone	7 (3.4%)	4 (2.2%)	3 (11.5%)	0.011
Dilatation	2 (0.8%)	1 (0.5%)	1 (3.8%)	ns
Pyelonephritis	1 (0.4%)	0 (0%)	1 (3.8%)	ns
Normal	193 (94.6%)	172 (96.6%)	21 (80.8%)	ns

Abbreviations: SD, standard deviation.

Overall, 92.8% of patients were treated with fluoroquinolones. The mean duration of treatment was 22.9 ± 6.6 days (18.9 ± 7.1 days for group 1 and 23.5 ± 6.3 days for group 2, *p <* 0.001). None of the patients failed to respond to the treatment. Men returned with apyrexia in mean 2.7 days ± 1.6 days. Only three men (one in group 1 and two in group 2) required hospitalization during follow up. No difference was found between groups. Men with suspected pyelonephritis did not evolve differently from those with suspected prostatitis. Monitoring and treatment of men with febrile urinary tract infection does not seem to depend on the existence of a suspected pyelonephritis suspected after the presence of a lumbar pain.

### Discussion

Herein, we present one of the largest studies published on febrile urinary infection in men. Clinical, laboratory and radiological data were studied, and we report many useful concepts for the diagnosis and treatment of acute prostatitis and acute pyelonephritis.

The clinical characteristics of acute prostatitis are poorly described in the published literature [2,3]. The majority of the literature focuses on post-biopsy prostatitis [4,5,6]. Flank pain is frequently described (from 5.1% in our study to 25%, two without any pyelonephritis), and prostatitis can be misdiagnosed initially as flank pain is interpreted as a clinical sign of pyelonephritis. Patients frequently describe urinary symptoms; however, the reported incidence for urinary symptoms is influenced by the specialty of the practitioner [3]. A total of 86% of patients questioned by a urologist for an acute prostatitis had urinary symptoms, while only 50% reported urinary symptoms if seen in geriatrics or internal medicine [3]. However, the population admitted to urology departments may have a higher incidence of bladder disorders than populations handled by other specialties. Only 63% of patients with AP in geriatric departments suffer from fever *versus* more than 80% in other departments (100% in our study). We could argue that the diagnosis of AP was suggested by geriatricians because of positive bacterial cultures in men with indwelling catheters. DREs suggesting AP are not routinely described (39% for infectious disease specialists to 77% for urologists) [3]. In our study, only 4.4% of DRE were painful. DRE were not routinely performed by GPs in our study, as the initial algorithm found that the predictive value of DRE in acute situations strongly suggest AP to be poor. Unfortunately, we did not focus on past history of urinary tract infection (UTI). Etienne *et al.* reported that approximately 37% of men with AP report previous UTI [3]. In our study we found that patients with flank pain had fewer previous episodes of urinary tract infection without arguing for pyelonephritis or prostatitis.

C reactive protein (CRP) and leukocyte levels were elevated in 96% and 74% of men with AP, respectively [2]. We found similar results that did not correlate with the symptoms or the severity of UTI and without difference between AP or pyelonephritis. Analyzing these results, we suggest that the predictive value of CRP and leukocyte levels is low in the diagnosis of UTI; its value should be more precisely determined.

Blood cultures were not available in our study. In a previously published study, blood cultures were analyzed for acute prostatitis patients [7]. Blood cultures were positive for 21% of men and contributed to the microbiological diagnosis for 5% of the patients. Guidelines are not trivial for that purpose; these data suggest that cases of uncomplicated UTI, AP or pyelonephritis would not benefit from routine blood cultures. However, urine cultures are recommended, and the bacterial results are similar between studies.

In a previous study, urine dipsticks were used to test 422 men with urinary infections [8]. The authors concluded that the positive predictive value of a positive nitrite test was 96% and that the addition of the results of the leukocyte esterase test did not improve the diagnostic accuracy of the nitrite test. We agree with these conclusions, and we would like to emphasize the poor negative predictive value of urine dipsticks in men UTIs.

Post-voiding residual volumes were not different between groups in our study. In the Etienne study, only 14% of patients followed by an infectious disease specialist had post-voiding residual volume measurements done, and 21% of patients had postvoiding residual volume measurements done if followed by a urologist [3]. This result was not precise in Auzanneau’s study, in which 49% had a renal or bladder ultrasound [2]. We did not find any additional value of performing a renal or a prostatic ultrasound, except for stone diagnosis in men with flank pain. Based on all of these findings, we can argue that the only imaging technique that should be performed on men with suspected AP is a bladder ultrasound to measure the postvoiding residual volume and a renal ultrasound if men complain from spontaneous or provoked flank pains. The algorithm used informed us to perform prostate ultrasound if the patient remains febrile at day 3 after antibiotics in order to diagnose an abscess. None of the patients needed prostate ultrasound.

To our knowledge, this is one of the largest studies analyzing a substantial amount of data in an important population of men with febrile UTI. Data were collected prospectively and analyzed retrospectively. The network increases the follow up of patients with routine phone calls and specialist visits if the evolution of UTI is unfavorable or if the patient experiences recurrent episodes. The data examined in this study emphasized that the laboratory tests or radiological exams should be performed accurately. The national and international infectious disease committee should take into account these results to provide new recommendations for the diagnosis and treatment of men UTIs. The question about the diffenciation between AP and prostatitis remains unsolved. We can argue that no difference was found between men with flank pain and men without flank pain in term of clinical or biological data. Stones were, however, more frequently found on ultrasound if men complain of flank pain.

## 4. Conclusions

This study, conducted using data from patients with febrile UTI, revealed that the most common clinical features are fever with urinary discomfort. No differences were found between men with or without flank pain, except for stones, which were more frequently diagnosed on ultrasound if men complained of flank pains. Laboratory test results and radiological features had a poor predictive value. Men with suspected pyelonephritis did not evolve differently from those with suspected prostatitis. Monitoring and treatment of men with febrile urinary tract infection does not seem to depend on the existence of a suspected pyelonephritis suspected after the presence of a lumbar pain. The results of this study will be very useful for the development of future guidelines concerning the management of febrile UTI in men. Ambulatory management of febrile UTI is feasible and safe, requiring an efficient network for patient surveillance.
